# IGF signaling in muscle degenerative diseases

**DOI:** 10.18632/aging.100625

**Published:** 2013-12-26

**Authors:** Kelly Hyunju Oh, Hongkyun Kim

**Affiliations:** Department of Cell Biology and Anatomy, The Chicago Medical School, Rosalind Franklin University of Medicine and Science, North Chicago, IL 60064

Evolutionarily conserved signaling pathways and transcription factors that sense physiological and environmental states regulate aging processes. Lifespan extension was achieved by the modulation of these pathways in yeast, worms, flies and mice. Remarkably, slowing aging processes resulted in the amelioration of age-dependent degenerative diseases (reviewed by Kenyon, Nature, 2010, 464: 504). Our recent study (Oh, PNAS, 2013, 110: 19024) shows that lifespan-extending signaling pathways may provide opportunities for therapeutic interventions of muscle degenerative diseases.

Muscular dystrophies are a heterogeneous group of muscle degenerative diseases. The most prevalent form of muscular dystrophies is Duchenne muscular dystrophy (DMD), which is caused by mutations in the dystrophin gene. Dystrophin connects intracellular structural components to the extracellular matrix, and its loss causes cellular damage and consequent myofiber necrosis. While intense efforts have been made to restore dystrophin expression through various methods, including gene transfer and exon skipping, without much success, the mechanism by which the loss of dystrophin function causes muscle cell death has not received much attention.

*Caenorhabditis elegans (C. elegans)* is an invertebrate genetic model organism. Dystrophin and its associated proteins and major signaling and cell death pathways are highly conserved from worms to humans. In our recent study, we have established a *C. elegans* model of dystrophinopathy. Mutations in the dystrophin gene (*dys-1*) result in muscle cell death in an age-dependent manner. *dys-1* mutant animals lost locomotory function earlier than wild-type animals, and their lifespan was shorter. *dys-1* mutants also exhibited morphological and structural defects. A muscle structure that attaches thin filaments to the muscle membrane was lost in *dys-1* mutants. Muscle nuclei and mitochondria were disintegrated in *dys-1* mutants. This array of phenotypic defects is not apparent in young adults; however, as the mutant animals get older, this array becomes obvious and exacerbated. In addition to such age-dependent severity, the affected muscle cells were random in their positions in the body and in their developmental lineage, even though all of the muscle cells lacked dystrophin. Similarly, in humans, the onset and progression of muscle degeneration and the muscle groups affected are highly variable, even among siblings with the same mutation in the dystrophin gene. Hence, we concluded that although muscle cell death is caused by the loss of dystrophin, age-dependent, intrinsically variable cellular environments influence the onset, severity and progression of the development of muscle cell death. We hypothesize that these cellular environments are the *modulators of susceptibility* and will be excellent therapeutic targets to thwart muscle cell death.

We reasoned that cellular stress responses would be the modulators of susceptibility because stress proteins and cellular calcium levels are elevated in the muscles of human DMD patients and mammalian models. The loss of dystrophin in *C. elegans* increases muscle excitability and contractility in an activity-dependent manner. The over-excitation of motoneurons leads to the disruption of calcium and protein homeostasis in *C. elegans* muscle cells. Therefore, we hypothesized that one of the cellular consequences of the loss of dystrophin could be the disruption of proteostasis. We tested this hypothesis using a polyglutamine protein aggregation model, where polyQ35 proteins in muscles form aggregates in an age-dependent manner and the onset and severity of polyQ35 aggregation serve as a proxy for protein homeostasis (Garcia SM et al. Genes Dev, 2007, 21:3006). Indeed, we found that polyQ35 forms aggregates earlier in *dys-1* mutants than in the wild type animals, indicating that the loss of dystrophin leads to the disruption of protein homeostasis. Compromised protein-folding capacity can lead to the misfolding and dysfunction of structurally unstable proteins that are tolerated under normal conditions. This phenomenon may contribute to the pathology of dystrophinopathy.

Our conclusion that muscle cell death in *dys-1* mutants is determined by cellular environments that are variable and age-dependent led us to reason that a sustained, rejuvenating cellular environment may provide protection from muscle cell death. Reductions in IGF signaling enhance stress resistance, delay inherent protein aggregation, and extend lifespan. Indeed, we found that reducing IGF signaling prevented muscle cell death in the *dys-1* mutant in a DAF-16 (homolog of FOXO) dependent manner. Consistent with our finding, the chronic activation of AMPK (AMP-activated protein kinase), which is a FOXO activator, was shown to increase sarcolemmal integrity in mdx mice (the mouse model of DMD) (Ljubicic V. et al. Human Mol Genetics, 2011, 20:3478). Rapamycin treatment, which inhibits the mTORC1 (the mammalian target of rapamycin complex 1), a protein complex down-regulated by FOXO under stress conditions, was shown to reduce muscle necrosis and fibrosis in the mouse model of Duchenne muscular dystrophy (Eghtesad S, Mol Medicine, 2011, 17:917).

Many important questions remain to be answered. What is the trigger of cell death? How does the collapse of proteostasis contribute to the cell death pathway in muscle cells? Which component of the IGF signaling pathways is critical for the prevention of cell death? Are there other signaling pathways that can thwart cell death? The answers to these questions will be the foundation for discovering new therapeutic interventions and biomarkers for Duchenne muscular dystrophy.

